# Addition of GM-CSF to trastuzumab stabilises disease in trastuzumab-resistant HER2+ metastatic breast cancer patients

**DOI:** 10.1038/sj.bjc.6605918

**Published:** 2010-09-28

**Authors:** Y C Cheng, V Valero, M L Davis, M C Green, A M Gonzalez-Angulo, R L Theriault, J L Murray, G N Hortobagyi, N T Ueno

**Affiliations:** 1 Division of Neoplastic Diseases and Related Disorders, Department of Medicine, Medical College of Wisconsin, Milwaukee, WI, USA; 2 Department of Stem Cell Transplantation and Cellular Therapy, The University of Texas MD Anderson Cancer Center, 1515 Holcombe Boulevard, Unit 448, Houston, TX, USA; 3 Breast Cancer Translational Research Laboratory, Department of Breast Medical Oncology, The University of Texas MD Anderson Cancer Center, 1515 Holcombe Boulevard, Unit 1354, Houston, TX 77030, USA

**Keywords:** granulocyte-macrophage colony-stimulating factor, HER2, metastatic breast cancer, trastuzumab

## Abstract

**Background:**

One of the proposed mechanisms of trastuzumab-induced regression of human epidermal growth factor receptor 2-positive (HER2+) tumours includes facilitation of antibody-dependent cell-mediated cytotoxicity (ADCC). Granulocyte-macrophage colony-stimulating factor (GM-CSF) mediates ADCC. We presented our pilot study of adding GM-CSF to trastuzumab in patients with trastuzumab-resistant HER2+ metastatic breast cancer.

**Methods:**

Patients with HER2+ metastatic breast cancer that progressed after trastuzumab +/− chemotherapy were continued on trastuzumab 2 mg kg^–1^ intravenous weekly and GM-CSF 250 *μ*g m^–2^ subcutaneous daily. Patients were assessed for response every 8 weeks. Treatment was continued until disease progression or intolerable toxicity.

**Results:**

Seventeen patients were evaluable (median age 48 years, range 27–75 years). The median number of metastatic sites was 2 (range 1–3); the most common site was the liver (*n*=10). The median number of prior regimens for metastatic disease was 2 (range 1–5). No objective disease response was observed, but five patients (29%) had stable disease for a median duration of 15.8 (range 10–53.9) weeks. The most common adverse event was rash at the injection site. No grade 4 or irreversible adverse event was seen.

**Conclusion:**

The addition of GM-CSF to trastuzumab alone had a modest clinical benefit and acceptable safety profile in heavily pretreated patients with trastuzumab-resistant HER2+ metastatic breast cancer.

Metastatic breast cancer is generally considered incurable by standard chemotherapy. Nevertheless, it is a chemosensitive disease. Among patients treated with chemotherapy, median survival is 24 months, and 2–5% patients have disease-free survival longer than 5 years. Multiple prognostic and predictive factors determine the course of the disease and the response to systemic treatment. One of these factors is expression of the human epidermal growth factor receptor 2 (HER2), which is overexpressed in ∼20% of breast cancers (Slamon *et al*, 2001).

The *HER2* oncogene is a member of the HER family of tyrosine kinase receptors. Amplification of *HER2* results in overexpression of the HER2 receptor, which correlates with several negative prognostic variables, including oestrogen receptor-negative status, high S-phase fraction, positive nodal status, mutated *p53*, and high nuclear grade (Sjögren *et al*, 1998). Overexpression of the HER2 receptor in turn results in relative resistance to endocrine therapy (Atalay *et al*, 2003) and is correlated with an aggressive form of breast cancer and significantly shorter disease-free and overall survival (Press *et al*, 1993; Seshadri *et al*, 1993; Ravdin and Chamness, 1995; Cobleigh *et al*, 1999; Slamon *et al*, 1987). As overexpression of HER2 receptor is such an important prognostic and predictive factor (Pietras *et al*, 1995), targeting this receptor with tumour-specific passive and active immunotherapeutic treatments is a rational strategy.

The humanised monoclonal antibody trastuzumab was developed as a therapy targeted against HER2 receptor. In patients with HER2-positive (HER2+) metastatic breast cancer, response rates to trastuzumab monotherapy range from 12 to 34%, median duration of response is 9 months (Cobleigh *et al*, 1999; Nahta *et al*, 2004). Concomitant trastuzumab and chemotherapy are synergistic and have resulted in better response rates, time to disease progression, and overall survival than chemotherapy alone or trastuzumab monotherapy. Therefore, concomitant trastuzumab and chemotherapy is considered a standard of care in HER2+ metastatic breast cancer (Seidman *et al*, 2001; Slamon *et al*, 2001; Esteva *et al*, 2002; Stein *et al*, 2004; Marty *et al*, 2005).

Although the mechanisms by which trastuzumab induces regression of HER2+ tumours are not known definitively, proposed mechanisms include potentiation of chemotherapy (Pegram *et al*, 1999), inhibition of tumour cell proliferation (Baselga *et al*, 1998; Sliwkowski *et al*, 1999), and facilitation of immune function through antibody-dependent cell-mediated cytotoxicity (ADCC) (Lewis *et al*, 1993; Sliwkowski *et al*, 1999; Repka *et al*, 2003). Thus, ADCC appears to be one of the most important immune effector functions. Cytokines such as granulocyte-macrophage colony-stimulating factor (GM-CSF) may augment ADCC by direct activation of immune cells or by enhancement of tumour-associated antigens on tumour cells (Sondel and Hank, 2001).

We hypothesised that adding GM-CSF to trastuzumab would overcome trastuzumab resistance via enhancing ADCC. We hereby present the results of our pilot study assessing the feasibility, safety profile, and efficacy of adding GM-CSF to trastuzumab in women with trastuzumab-resistant metastatic breast cancer.

## Patients and methods

### Trial design

All patients provided written informed consent prior to participating in the pilot study, and the study was reviewed and approved by the Institutional Review Board at The University of Texas MD Anderson Cancer Center. Eligible patients were women with metastatic breast cancer who had HER2-overexpressing disease (HER2 3+ by immunohistochemical staining or amplification by fluorescence *in situ* hybridisation) that was progressing after treatment with at least one cycle of trastuzumab with or without chemotherapy. Patients were required to have an Eastern Cooperative Oncology Group performance status score of 0 or 1, an adequate haematological profile, and adequate liver, kidney, and heart function. Patients also were required to have measurable metastatic disease. Patients with only bone disease, only leptomeningeal disease, or only malignant pleural effusion were not eligible.

### Treatment

Trastuzumab was intravenously given as follows: a 4 mg kg^–1^ loading dose followed by 2 mg kg^–1^ every week for 4 weeks (one cycle). A loading dose was not necessary if patients received trastuzumab within 2 weeks before the start of study treatment. Subcutaneous GM-CSF was given at 250 *μ*g m^–2^ daily until the absolute neutrophil count was >10 000 mm^–3^, then was given every other day to maintain the absolute neutrophil count below 10 000 mm^–3^. When GM-CSF and trastuzumab were given on the same day, GM-CSF was given before trastuzumab infusion. Patients underwent restaging every 8 weeks (two cycles). Granulocyte-macrophage colony-stimulating factor and trastuzumab were continued until disease progression or intolerable toxic effects. The primary end points of the trial were tumour response (including stable disease) and time to progression. Treatment-related toxicity was assessed according to the National Cancer Institute Common Toxicity Criteria version 2.0. Tumour response was assessed according to the Response Evaluation Criteria in Solid Tumours system.

### Statistical consideration

A maximum of 18 patients will be entered in the study. A 15% (three patients) improved tumour response (including stable disease) rate in this patient population would provide an impetus towards further investigation of this treatment, including measurement of ADCC.

## Results

Eighteen patients with progressive HER2+ metastatic breast cancer were eligible, and 17 were evaluable (median age 48 years, range 27–75 years) (Table 1). One patient did not complete the first cycle because of a protocol violation. Among the 17 evaluable patients, 9 had hormone receptor-positive disease. The median number of sites of metastasis was 2 (range 1–3). The most common site of metastasis was the liver (*n*=10). The median number of trastuzumab-containing regimens for metastatic disease was 2 (range 1–5). One patient developed rapidly progressive disease 2 weeks after the start of study therapy and died soon after. Sixteen patients received treatment for at least 8 weeks (two cycles) until disease progression. No disease response was observed, but five patients (29%) had stable disease more than 8 weeks. The median duration was 15.8 weeks (range 10–53.9 weeks). Three of the five patients had hormone receptor-negative disease and four of them had visceral organ involvement. Thirteen patients had grade 1 adverse events; six patients had grade 2 adverse events; and two patients had grade 3 adverse events (fatigue, muscle aches, and paraesthesia). The most common adverse events, in decreasing order of frequency, were rash at the GM-CSF injection site, skin rash, fatigue, and muscle aches (Table 2). No grade 4 or irreversible adverse event was seen.

## Discussion

Several proposed mechanisms of trastuzumab resistance included downregulation of HER2, upregulation of expression of *PTEN* gene or insulin-like growth factor receptor I gene, and also expression of a truncated form of HER2 receptor – p95HER2 (Lu *et al*, 2001; Scaltriti *et al*, 2007). Our pilot study has demonstrated the potential for therapeutic synergy when trastuzumab is combined with GM-CSF in patients with trastuzumab-resistant HER2+ metastatic breast cancer. The addition of GM-CSF to trastuzumab alone provided clinical benefit in 29% of heavily pretreated patients without causing any grade 4 adverse events.

We tested a different approach to overcoming tumour resistance to trastuzumab: enhancing the effect of trastuzumab through addition of cytokines. One of the antitumour effects of trastuzumab is through the action of innate effector mechanisms, such as ADCC (Drebin *et al*, 1988; Kim *et al*, 2002; Spiridon *et al*, 2002). As a mediator of ADCC, trastuzumab is detected as an abnormality on the HER2 receptor of tumour cells by natural killer (NK) cells, which in turn secrete cytokines and subsequently lead to tumour cell death (Lewis *et al*, 1993; Sliwkowski *et al*, 1999). The GM-CSF is commonly used to augment immune response by increases antigen presentation of monocytes and macrocytes, enhances CD20 expression, stimulates the effector function of myeloid cells (i.e., neutrophils, macrophages, NK cells, and dendritic cells), and enhances cell-mediated immunity (Dranoff, 2004; Niitsu *et al*, 2004; Olivieri *et al*, 2005). The GM-CSF also mediates ADCC via stimulation of macrophages (Kushner and Cheung, 1989; Erbe *et al*, 1990; Liesveld *et al*, 1991; Tarr, 1996), and the ability of GM-CSF to increase the production of granulocytes and mononuclear cells, as well as to enhance their cytotoxic activities against tumour cells, is well documented (Kushner and Cheung, 1989; Erbe *et al*, 1990; Liesveld *et al*, 1991; Ragnhammar *et al*, 1992; Tarr, 1996; Yu *et al*, 1997). The GM-CSF also can affect the migration of granulocytes (Gasson *et al*, 1984; Barker *et al*, 1991), resulting in their increased accumulation at tumour sites (Tseng *et al*, 1999).

Currently, GM-CSF is most often used in cancer treatment as a stimulant of leukocyte production to protect against infection (Jones *et al*, 1996; Beveridge and Miller, 1998). However, as GM-CSF can augment immune effector cell functions (Kushner and Cheung, 1989; Erbe *et al*, 1990; Liesveld *et al*, 1991; Tarr, 1996), it also may enhance the therapeutic effect of monoclonal antibodies, such as trastuzumab.

Several experimental and clinical studies have demonstrated the antineoplastic effects of GM-CSF alone or in combination with cytokines and/or monoclonal antibodies (Ragnhammar, 1996). The GM-CSF has been shown to enhance anti-G_D2_-mediated ADCC by granulocytes in disease-free subjects and in patients with neuroblastoma (Yu *et al*, 1997; Batova *et al*, 1999). A recent pilot trial found that continuous, low-dose GM-CSF had substantial activity (objective response rate 37%) in heavily pretreated patients with either metastatic breast cancer or female genital tract cancer (Kurbacher *et al*, 2005). Enhancement of ADCC of human peripheral blood mononuclear cells by GM-CSF has been described (Grabstein *et al*, 1986; Thomassen *et al*, 1989), and GM-CSF in conjunction with monoclonal antibodies has been used in clinical trials for the treatment of colorectal carcinoma (Mellstedt *et al*, 1991; Ragnhammar *et al*, 1992) and neuroblastoma (Yu *et al*, 1997). Trial results found that GM-CSF augmented ADCC activity of mononuclear cells and granulocytes against both colorectal cancer cells and neuroblastoma; therapeutic efficacy was demonstrated in these trials (Mellstedt *et al*, 1991; Ragnhammar *et al*, 1992; Yu *et al*, 1997). In this study, we have initially designed to continue the trial in a phase II setting including measurement of the ADCC activities under the influence of GM-CSF and trastuzumab. However, we were not able to accrue patients further to the study due to other competing trials in our institution.

Although targeted therapies, such as combinations of trastuzumab and chemotherapy, have been widely investigated for the treatment of metastatic breast cancer, the role of cytokines, such as GM-CSF, as an immunological stimulant in combination with monoclonal antibodies, has been less well examined. Our pilot study has demonstrated the potential for therapeutic synergy with the combination of GM-CSF and trastuzumab. Administration of GM-CSF is simple, safe, and feasible. Although no disease response was seen in our pilot study, our finding that this simple approach stabilised breast cancer is clinically significant in this setting of metastatic disease. This trastuzumab plus GM-CSF regimen needs further evaluation in combination with chemotherapy or other biological agents in the management of metastatic breast cancer. Further, there is a need to determine whether ADCC activities are measured.

## Figures and Tables

**Table 1:** Patient characteristics^a^

*Number of patients*	
Total	18
Evaluable	17
	
Age in years, median (range)	48 (27–75)
	
*Initial disease stage*	
I	1
II	5
III	6
IV	5
	
*Hormone receptor status*	
Positive	9
Negative	8
	
HER2 receptor positive	18
Neoadjuvant chemotherapy	6
Anthracycline-containing regimen only	4
Anthracycline- and taxane-containing regimen	2
	
Adjuvant chemotherapy	9
Anthracycline-containing regimen only	2
Anthracycline- and taxane-containing regimen	3
Taxane-containing regimen only	4
	
Adjuvant radiation therapy	5
Adjuvant hormone therapy	3
Number of systemic regimens after metastasis, median (range)	2 (2–8)
Number of trastuzumab-containing regimens after metastasis, median (range)	2 (1–5)
Number of sites of metastasis, median (range)	2 (1–3)
	
*Sites of metastasis*	
Liver	10
Bone	8
Lymph nodes	6
Lung	5
Chest wall	2
Brain	1
Peritoneum	1

a Values are numbers of patients unless otherwise specified.

**Table 2:** Toxic effects of treatment

**Toxic effect**	**Total**	**Grade 1**	**Grade 2**	**Grade 3**
Fever	3	2	1	0
Nausea	2	1	2	0
Vomiting	1	0	1	0
Sore mouth	2	2	0	0
Diarrhoea	3	3	0	0
Constipation	1	1	0	0
Fatigue	5	5	2	1
Muscle pain	4	3	3	1
Numbness	1	1	1	1
Sore fingers/toes	1	1	0	0
Red eye	1	1	0	0
Rash at injection site	7	6	1	0
Skin rash	6	5	2	0
Itchy hands/feet	1	1	0	0
Headache	2	2	1	0
